# Empty virions in AAV8 vector preparations reduce transduction efficiency and may cause total viral particle dose-limiting side effects

**DOI:** 10.1038/mtm.2013.9

**Published:** 2014-01-29

**Authors:** Kai Gao, Mengxin Li, Li Zhong, Qin Su, Jia Li, Shaoyong Li, Ran He, Yu Zhang, Gregory Hendricks, Junzhi Wang, Guangping Gao

**Affiliations:** 1 Gene Therapy Center, University of Massachusetts Medical School, Worcester, Massachusetts, USA; 2 National Institutes for Food and Drug Control, Beijing, China; 3 Viral Vector Core, University of Massachusetts Medical School, Worcester, Massachusetts, USA; 4 Department of Microbiology and Physiology Systems, University of Massachusetts Medical School, Worcester, Massachusetts, USA; 5 Division of Hematology/Oncology, Department of Pediatrics, University of Massachusetts Medical School, Worcester, Massachusetts, USA; 6 Department of Oncology, Guizhou People’s Hospital, Guiyang, Guizhou, China; 7 Department of Cell and Developmental Biology, Electron Microscopy Core Facility, University of Massachusetts Medical School, Worcester, Massachusetts, USA; 8 Key Laboratory of Biotherapy, West China Hospital, Sichuan University, Chengdu, Sichuan, China

## Abstract

Empty virions are inadvertent by-products of recombinant adeno-associated virus (rAAV) packaging process, resulting in vector lots with mixtures of full and empty virions at variable ratios. Impact of empty virions on the efficiency and side effects of rAAV transduction has not been well characterized. Here, we generated partially and completely empty AAV8 virions, fully packaged rAAV8 lots, and mixtures of empty and fully packaged virions with variable ratios of empty virions. The aforementioned dosing formulations of rAAV8 expressing either cellular (*EGFP* (enhanced green fluorescent protein) or nuclear-targeted (n) *LacZ*) or secreted (human α1-antitrypsin (*hA1AT*)) reporter genes were intravenously injected into two different mouse strains, followed by analyses of transgene expressions and serum alanine aminotransferase (ALT) levels at different time points. We found that addition of empty particles to the fixed doses of rAAV8 preparations repressed liver transduction up to 64% (serum hA1AT) and 44% (nLacZ) in C57BL/6 mice, respectively. The similar trend in inhibiting EGFP expression together with concurrent elevations of serum ALT levels were observed in the BALB/c mice, indicating that empty particles may also exacerbate side effects of rAAV8 *EGFP* transduction. Our results suggest that removal of empty particles from rAAV preparations may improve efficacy and safety of AAV in clinical applications.

## Introduction

Adeno-associated virus (AAV), a small single-stranded DNA-containing nonpathogenic human parvovirus, is an efficient gene transfer vehicle for gene transfer to different tissues including liver, without apparent vector-related toxicities.^1–3^ Recombinant AAV (rAAV) has been clinically evaluated for *in vivo* gene therapy applications, including treatment of hemophilia.^4–9^ The rAAV serotype 2–mediated liver gene transfer for treatment of hemophilia in human resulted in a transient increase in hepatic enzymes and loss of transgene expression subsequently indicating activation of CD8^+^ T-cell responses against AAV2 capsids.^10,11^ The magnitude of such adaptive immune responses appeared to be dose and serotype of AAV capsid dependent, suggesting that a high liver-tropic and low immunity AAV vector may be needed for effective liver-directed gene therapy.^10–12^ Nonhuman primate–derived rAAV serotype 8 outperformed all other AAV serotypes^13^ in transducing hepatocytes *in vivo* and could be an ideal candidate for this purpose. Recently, combination of AAV8 capsid with self-complementary vector genome to target liver in another hemophilia B gene therapy trial indeed led to sufficient FIX transgene expression and improved bleeding phenotype.^14^ However, several patients with high doses of AAV8 vector delivery also had transient increases in transaminases associated with increased AAV capsid–specific T cells and decreased circulating hF.IX levels, although such a vector-related immunotoxicity seemed to be resolvable by anti-inflammation steroid regimens of prednisolone.^14,15^

Moreover, it is reported that clinical grade AAV vector lots may consist of mixtures of empty and full virions at variable ratios of empty virions (REVs) up to 90%, depending on purification methods.^16,17^ Therefore, it has been speculated that nonfunctional empty virions in clinical vector lots may reduce efficiency of therapeutic gene transduction in the liver by competing with the fully packaged therapeutic vector particles for receptor uptake, internalization, and intracellular trafficking; they may also exacerbate vector-related side effects. However, these hypotheses have not yet been formally investigated in *in vivo* animal studies.

Here, using the method described by Ayuso *et al*.,^18^ we generated AAV8 capsid preparations with partially empty (PE (60–90%)) and completely empty (CE (100%)) virions (PE and CE, respectively) as well as rAAV8 lots with >99% of fully packaged virions. We mixed the fully packaged rAAV8 vectors expressing cellular (i.e., *EGFP* (enhanced green fluorescent protein) or nuclear-targeted (n) *LacZ*) and secreted (human α1-antitrypsin (*hA1AT*)) reporter genes, respectively, with either CE or PE virions at different REVs (i.e., 25, 50, 75, and 90% of empty virions in total particles) to simulate different scenarios of REVs with real rAAV8 clinical lots. We then intravenously dosed adult male mice of two different strains (i.e., C57BL/6 and BALB/c) with the vector preparations. At different time points after injections, we analyzed the serum hA1AT levels (C57BL/6 mice, rAAV8 *hA1AT* groups only), EGFP (BALB/c), and nLacZ (C57BL/6) expression in the liver sections in the corresponding groups as well as serum alanine aminotransferase (ALT) levels in all groups of the treated mice. Our results revealed that as the percentages of empty virions increase within a certain range in the dosing vectors: (i) transgene expression decreases (as much as 70%) for all three reporter genes in the both strains of mice and (ii) serum ALT levels elevate (as much as threefold) in BALB/c mice treated with EGFP vector. Also, the empty capsids generated in the vector production/purification process appear to be more toxic than those produced in the absence of vector genome plasmid. In summary, our study substantiated the negative impact of the empty virions in dosing AAV vectors on the gene transfer efficiency and total viral particle dose-limiting side effects and highlighted the importance of removal empty particles from clinical grade rAAVs to further improve the efficacy and safety of rAAV gene therapy.

## Results

### Efficient removal of empty virions from rAAV8 vector lots by CsCl gradient centrifugation

**Figure 1:**
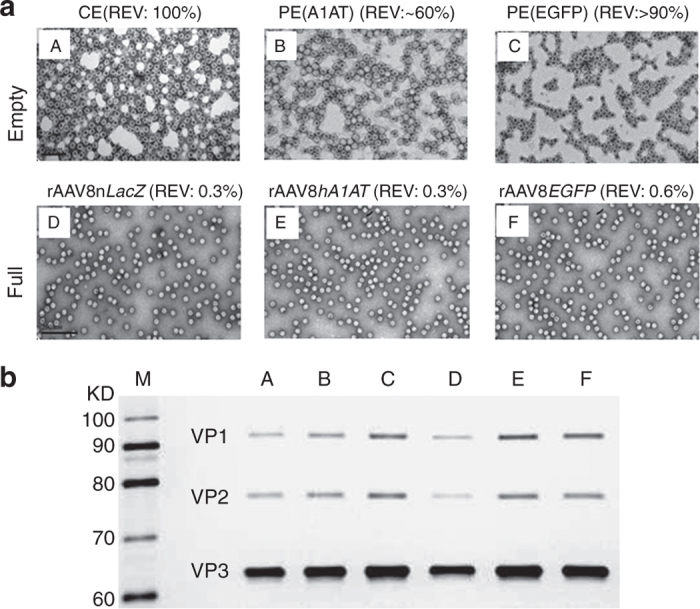
Efficient removal of empty virions from rAAV8 preparations by CsCl gradient centrifugation. (**a**) Shown are transmission electron microscopy images of rAAV8*nLacZ* (D) rAAV8*hA1AT* (E), and rAAV*EGFP* (F) vectors as well as completely empty (CE) virions (A) and partially empty (PE) virions from rAAV8*hA1AT* (B) or rAAV8*EGFP* (C) production/purification process. The ratio of empty virions was semiquantitatively determined by counting six representative fields using high-resolution electron microscopy. The viral particles with the dimpled (dark) center are the empty virions. Original magnification: ×92,000. (**b**) Equal amounts of all rAAV8 vector or empty virion samples (1 × 10^10^ viral particles) were analyzed by sliver-stained SDS-PAGE. EGFP, enhanced green fluorescent protein; nLacZ, nuclear-targeted LacZ; rAAV, recombinant adeno-associated virus; SDS-PAGE, sodium dodecyl sulfate–polyacrylamide gel electrophoresis.

To assess the efficiency of CsCl gradient sedimentation in removing empty virions from fully packaged rAAV particles, we used high-resolution transmission electron microscopy (EM) to examine morphology of negative-stained virions^19^ in CE (Figure 1a-A) and PE (Figure 1a-B, contaminated with virions containing rAAV*hA1AT* genomes and Figure 1a-C, contaminated with virions containing rAAV*EGFP* genomes) AAV8 capsids well as fully packaged rAAV8*nLacZ* (Figure 1a-D), rAAV8*hA1AT* (Figure 1a-E), and rAAV8*EGFP* (Figure 1a-F). It is worth pointing out that the PE AAV8 particles were derived from the empty virion fractions collected from the second CsCl gradient sedimentation in the purification processes for rAAV8*hA1AT* (Figure 1a-B) and rAAV*EGFP* (Figure 1a-C) respectively, whereas the CE AAV8 particles (Figure 1a-A) were produced by using AAV8 packaging plasmid and adenoviral helper gene plasmid only for 293 cell transfection. As shown in Figure 1a, while CE and PE particles primarily displayed donut-like shapes of virions without (Figure 1a-A, REV: 100%) or with variable amounts of fully packaged particles (Figure 1a-B, REV: ~60% and Figure 1a-C, REV: >90%), more than 99% of fully packaged virions were observed in all three lots of rAAV8 vectors (Figure 1a-D–E), which was confirmed with a semi-quantitative assessment by counting all empty and full virions in six representative fields at ~92,000× using high-resolution transmission EM (Figure 1b). To test the purity of all the viral preparations, equal amounts of viral particles (~1 × 10^10^ viral particles) of each sample were analyzed by sliver-stained sodium dodecyl sulfate–polyacrylamide gel electrophoresis, which showed that viral protein (VP)1, VP2, and VP3 capsid proteins were the only detectable protein components in all preparations (Figure 1b). Our results documented that the empty virions as well as other impurity proteins of cellular and transgene origins were efficiently removed from rAAV8 vector preparations by the CsCl gradient sedimentation method described by Ayuso *et al*.^18^ These highly purified AAV8 vectors and empty virions were next evaluated for transduction efficiency and side effects in mice for liver-directed gene transfer.

### Repression of rAAV8-mediated transgene expression in mouse liver by empty virions in the dosing vector formulations

It was reported that the tolerogenic response to systemic AAV-delivered antigens was observed in the liver of C57BL/6 but not BALB/c mice.^20–22^ On the other hand, lack of tolerance induction in BALB/c mice could lead to cytotoxic T lymphocyte–mediated clearance of transduced hepatocytes.^20–22^ These two strains of mice may serve as suitable models for investigating potential effects of empty AAV8 virions on rAAV8-mediated hepatocyte transduction and side effects *in vivo*, respectively.

**Figure 2:**
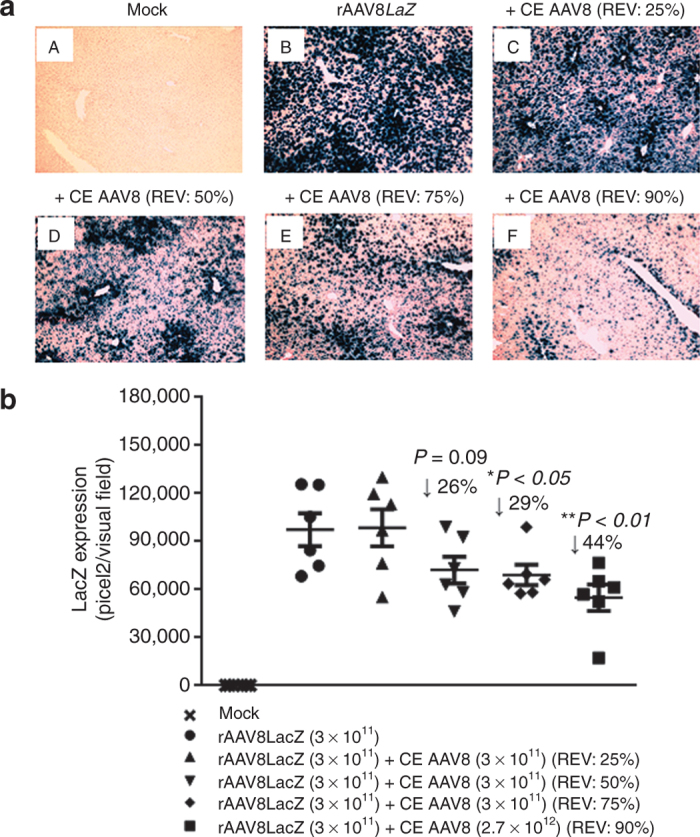
Repression of nLacZ liver transduction by increased ratios of empty adeno-associated virus (AAV) particles in C57BL/6 mice. (**a**) Mice were intravenously injected with rAAV8*nLacZ* vectors (3 × 10^11^ GCs/mouse) alone or mixed with AAV8 completely empty (CE) at variable ratios of empty virions (REVs: 25–90%) via tail vein. The liver sections were stained with X-Gal histochemically. Transgene expression was detected by light microscopy at 35-day postinjection. Original magnification: ×100. (**b**) Quantitative analyses of rAAV8 transduction efficiency. Images from six visual fields were analyzed quantitatively using ImageJ analysis software. Transgene expression was assessed as total area of blue staining (pixel)^2^ per visual field (mean ± SEM). Student’s *t*-test was used to compare test results with the group received rAAV8 alone, and the differences were determined to be statistically significant. *P < 0.05, **P < 0.01. GC, genome copy; nLacZ, nuclear-targeted LacZ; rAAV, recombinant AAV.

To simulate different clinical scenarios, we first mixed a fixed vector genome copy (GC) dose (3 × 10^11^ GCs) of highly purified rAAV8*nLacZ* vector consisting of >99% of fully packaged viral particles with none, (REV: 0%; Figure 2a-B), 1 × 10^11^ (REV: 25%; Figure 2a-C), 3 × 10^11^ (REV: 50%; Figure 2a-D), 9 × 10^11^ (REV: 75%; Figure 2a-E), and 2.7 × 10^12^ (REV: 90%; Figure 2a-F) viral particles of CE AAV8 virions as dosing vector formulations which were intravenously administrated to adult male C57BL/6 mice. The images of X-gal histochemically stained liver sections from the study animals harvested 5 weeks later revealed that the higher the REV, the stronger repression of nLacZ transduction (Figure 2a). Quantification of nLacZ transduction for each liver section confirmed the REV-dependent inhibition of rAAV8 transduction by CE particles; the inhibition became detectable at a REV of 50% and statistically significant at REVs of 75 and 90%. Such inhibitions led to 29% (REV: 75%; *P <* 0.05) and 44% (REV: 90%; *P <* 0.01) reductions in nLacZ transduction as compared with fully packaged rAAV8-nLacZ alone (Figure 2b).

**Figure 3:**
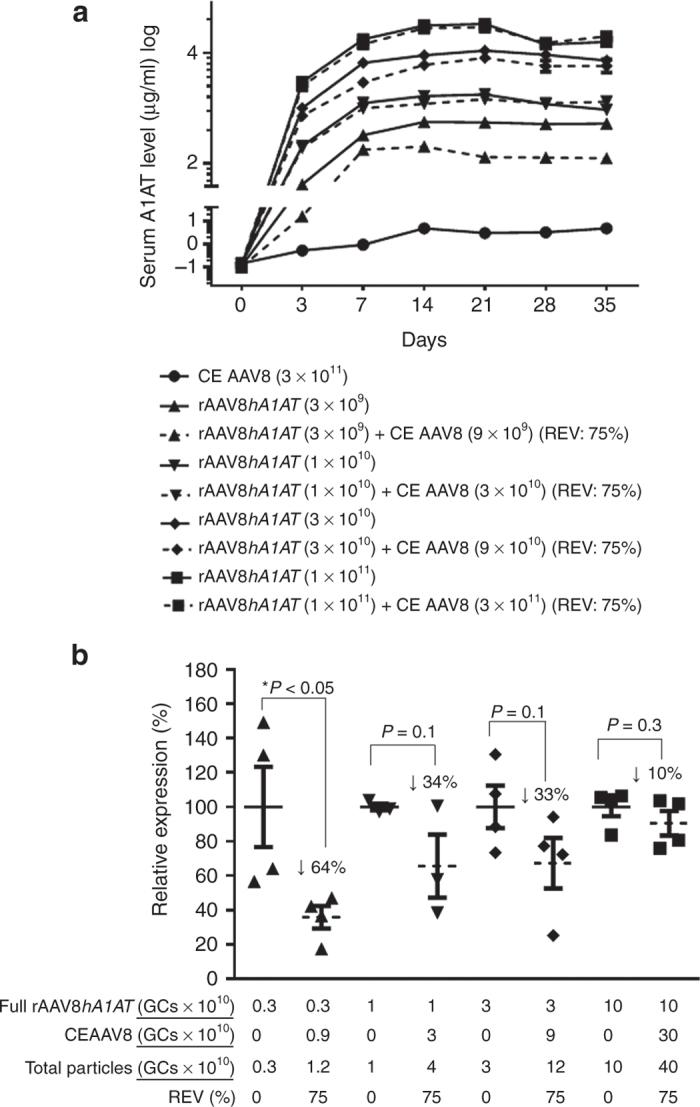
Human α-antitrypsin reporter gene expression from variable doses of rAAV8*hA1AT* with a fixed ratios of empty virion (REV) of AAV8 completely empty (CE) at different time points in C57BL/6 mice. **(a) ** Mouse sera were collected at different time points after intravenous injection of variable doses (1 × 10^9^ to 1 × 10^11^ GCs/mouse) of rAAV8*hA1AT* vectors with or without spiked-in CE AAV8 particles at a fixed REV of 75% (of total particles). The serum hA1AT levels were detected by enzyme-linked immunosorbent assay. (**b**) Relative serum hA1AT expression of animal groups received different rAAV8 dosing formulations as compared with the groups treated with various dose of fully packaged rAAV8 alone at 2-week postinjection. Student’s *t*-test was used for comparing the experimental results with those from the groups with various dose of fully packaged rAAV8 alone, and the differences were determined to be statistically significant *P < 0.05. GC, genome copy; rAAV, recombinant adeno-associated virus.

In an attempt to further characterize repression of rAAV8 liver transduction by AAV8 CE particles, we performed another experiment in C57BL/6 mice with a fully packaged rAAV vector expressing a quantitative secreted reporter gene *hA1AT* at variable GC doses (i.e., 3 × 10^9^, 1 × 10^10^, 3 × 10^10^, and 1 × 10^11^ GCs) and a fixed REV at 75%. This experiment generated the following findings. First, at all GC doses tested, only the lowest dose (3 × 10^9^) seemed to show significant repression of hA1AT transduction by AAV8 CE particles at all six time points tested (Figure 3a). Second, we compared hA1AT expressions between the groups of mice received fully packaged rAAV8 only and fully packaged rAAV8 mixed with AAV8 CE particles at a REV of 75% for all four different vector GC doses at the 2-week time point. Although only at the GC dose of 3 × 10^9^, a 64% (*P <* 0.05) inhibition of hA1AT expression by AAV8 CE particles became statically significant, 33–34% reductions of hA1AT expression were also noticeable in the 1 × 10^10^ and 3 × 10^10^ GC dose groups (Figure 3b).

**Figure 4:**
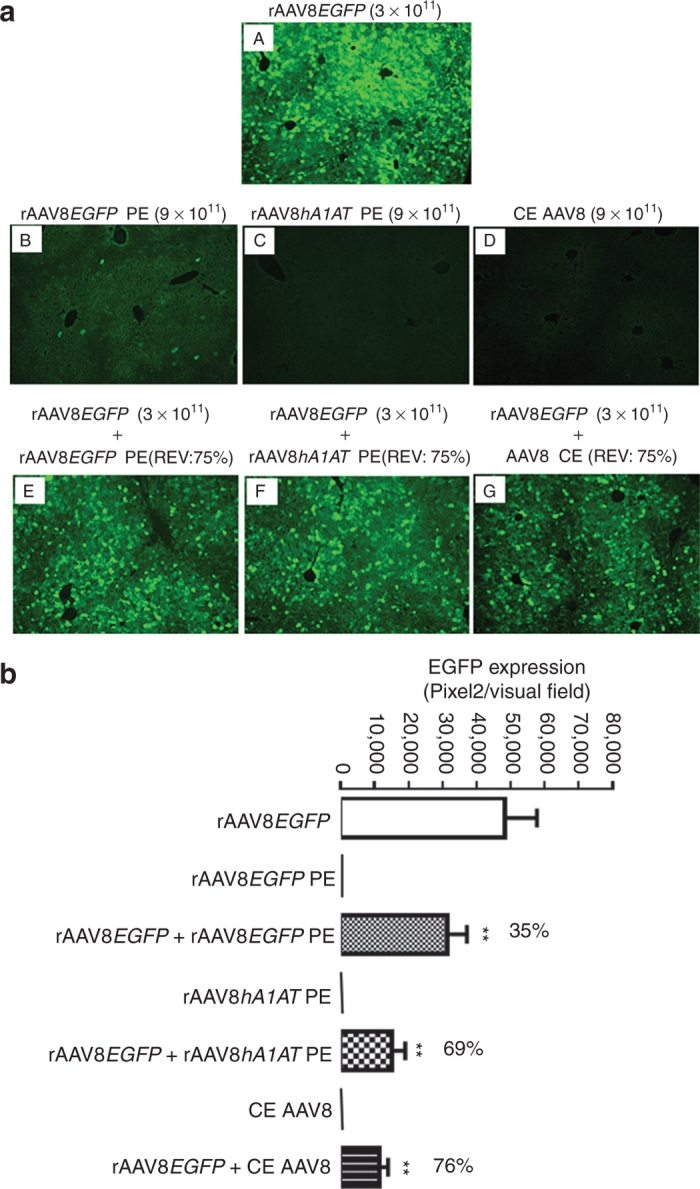
Repression of rAAV8*EGFP* liver transduction by completely empty (CE) and partially empty (PE) AAV8 particles in BALB/c mice. (**a**) Adult male BALB/c mice were intravenously injected with rAAV8*EGFP* vectors (3 × 10^11^ GCs/mouse) alone or mixed with empty AAV8 particles (9 × 10^11^ GCs/mouse) from three different sources at a fixed ratio of empty virion (REV: 75%) via tail vein. The liver sections were fixed, and transgene expression was detected by fluorescence microscopy at 4-week postinjection. Original magnification: ×100. (**b**) Quantitative analyses of rAAV8*EGFP* transduction efficiency. Images from six visual fields were analyzed quantitatively using ImageJ analysis software. Transgene expression was assessed as total area of green fluorescence (pixel)^2^ per visual field (mean ± SEM). Analysis of variance was used to compare test results with those from the group with rAAV8*EGFP* alone, and the differences were determined to be statistically significant. *P < 0.05, **P < 0.01. GC, genome copy; rAAV, recombinant adeno-associated virus.

We then repeated our experiment with a fixed GC dose of 3 × 10^11^ of rAAV8 and a fixed REV of 75% in BALB/c mice which are less tolerogenic to rAAV gene transfer to the liver. In this case, we used EGFP as a cellular reporter gene which is known to be immunotoxic in the BALB/c mouse liver.^21^ In addition, considering the fact that the only source of empty capsid contamination in clinical vector lots is from the vector production/purification process, we added two more groups of BALB/c mice to receive fully packaged rAAV8*EGFP* with spiked-in PE AAV8 capsids prepared as described earlier. As shown in Figure 4a, compared with rAAV8*EGFP* vectors alone (Figure 4a-A), EGFP expression in the livers at 4-week postinjection was clearly repressed by addition of the empty virions from three different sources (i.e., PE from rAAV8*EGFP* production/purification process, Figure 4a-E; PE from rAAV8*hA1AT* production/purification process, Figure 4a-F; and CE of AAV8, Figure 4a-G). As expected, while scattered EGFP-positive hepatocytes were visible in the livers of the animals received PE particles from rAAV8*EGFP* production/purification process (Figure 4a-B), no EGFP signal was detected in the liver sections from the groups received PE particles from rAAV8*hA1AT* production/purification process (Figure 4a-C) and CE AAV8 particles (Figure 4a-D). Quantification of EGFP expression on liver sections of the study animals at 4-week time point further documented the significant repression of rAAV8*EGFP* transduction in mouse livers by empty viral particles regardless of the source of the empty particles; addition of 9 × 10^11^ particles each of PE virions derived from rAAV*EGFP* and rAAV*hA1AT* production/purification processes as well as CE AAV8 virions to 3 × 10^11^ GCs of fully packaged rAAV8*EGFP* all resulted in a statistically significant 35, 69, and 76% of reduction of EGFP expression, respectively (Figure 4b).

Taken together, empty AAV8 particles, regardless of their origins, in rAAV8 vector dosing preparations repressed different reporter gene transduction in mouse liver. Such transduction inhibition was more pronounced in rAAV-mediated EGFP gene transfer to the liver of BALB/c mice.

### Exacerbation of the side effects of rAAV8*EGFP* liver transduction by empty AAV8 virions in BALB/c mice

As aforementioned, one of the possible mechanisms for the interference of rAAV*EGFP* transduction by empty AAV8 virions could be the increased immunological burden from excessive empty viral capsids, which may exacerbate vector-related side effects in mouse liver. To test this hypothesis, we followed serum levels of ALT, a highly informative biomarker for liver damage, which could be caused by vector-related side effects, at days 3, 7, 14, 21, 25, and 35 after vector infusions in both C57BL/6 and BALB/c mice that were used for the experiments as described above. Consistent with published studies,^20,22^ C57BL/6 mice were tolerogenic to rAAV gene delivery to the liver, and no significant ALT elevations were noticed in all experimental groups at all the time points, regardless of different reporter genes and REVs (data not shown).

**Figure 5:**
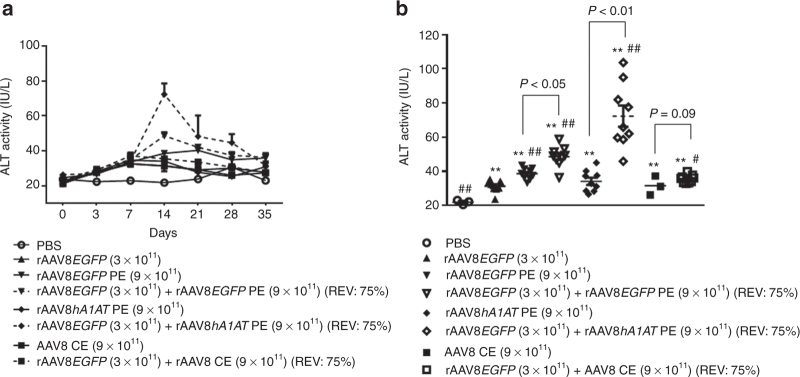
Exacerbate liver transaminitis in BALB/c mice by increased ratios of empty adeno-associated virus (AAV) particles in rAAV8*EGFP* dosing formulations. Mouse sera were collected at different time points after vector perfusion. The serum alanine aminotransferase (ALT) levels were detected by ALT detection kits. The time courses of (**a**) the ALT levels and (**b**) comparison of ALT levels of different study groups are presented. Analysis of variance was used for comparing the experimental results with those from the groups with phosphate-buffered saline (PBS) or rAAV8 or AAV8 empty particles alone and was determined to be statistically significant. *P < 0.05, **P < 0.01 versus PBS; ^#^*P* < 0.05, ^##^*P* < 0.01 versus rAAV*EGFP* alone. Finally, the groups of *rAAV8EGFP* vector mixed with different AAV8 empty particles were compared with the corresponding AAV8 empty particle alone, respectively, and the *P* value for each paired comparison is presented. rAAV, recombinant AAV.

On the other hand, our data also recapitulated previously reported side effects of rAAV transduction in BALB/c mice.^15,20,21,23^ First, although ALT elevations in the group treated with fully packaged rAAV8*EGFP* only were not as dramatic as what were previously reported,^20–22^ which may be attributed to possible differences in purification methods, serum ALT elevations were indeed observed as early as 1 week after vector injection, peaked at day 14 and not declined to those in the phosphate-buffered saline control group until the third week (Figure 5a). Second, infusions of 9 × 10^11^ CE and PE AAV8 particles to BALB/c mice all caused elevations of serum ALT levels in a pattern similar to those seen in 3 × 10^11^ GCs of fully packaged rAAV8*EGFP* only groups (Figure 5a), suggesting potential side effects of AAV8 capsids. Of note, addition of PE AAV8 particles derived from either rAAV*EGFP* or rAAV*hA1AT* production/purification process but not CE AAV8 particles at a REV of 75% to 3 × 10^11^ GCs of fully packaged rAAV8*EGFP* further aggravated ALT transaminitis, which was not resolved until 5 weeks after vector infusion (Figure 5a). A zoomed-in analysis of the ALT data at the 2-week time point, the peak of vector-caused transaminitis, confirmed that (i) administration of AAV particles, fully packaged, PE, and CE, all invoked slight but significant ALT elevations as compared with the phosphate-buffered saline group (all *P <* 0.01) and (ii) at a REV of 75%, exacerbation of vector-induced transaminitis was seen with empty AAV8 particles derived from rAAV8 production processes but not from the process without vector plasmid (Figure 5b). In other words, the empty capsids generated in the vector production/purification process appear to be more immunogenic than those produced in the absence of vector genome plasmid.

## Discussion

rAAV8 vector has shown great promise in the liver-directed clinical gene therapy of hemophilia B.^14^ However, rAAV8*hFIX* lots that have currently been administrated to hemophilia patients are tinted with up to 90% of carried-over nontherapeutic but rather potentially immunotoxic empty AAV8 capsids from rAAV production process.^16,17^ Efficient removal of empty particles from vector lots is a major challenge for good manufacturing practice–compatible rAAV production. The REV of a rAAV lot is not considered as a product release criterion for clinical applications. This raises major concerns over the efficacy and safety of clinical vector lots manufactured by the current good manufacturing practice rAAV production process.

In an attempt to address those concerns, we performed the present study. Our findings substantiated the repression of rAAV8 liver transduction in both C57BL/6 and BALB/c mice (Figures 2–4) and exacerbation of vector-related liver transaminitis in BALB/c mice (Figure 5) by empty AAV8 particles in the rAAV8 dosing formulations. Our findings further revealed that even though all empty AAV particles, regardless how they were generated, were inhibitory to rAAV8 liver transduction, only those empty AAV8 particles that were derived from rAAV8 production/purification processes appeared to worsen vector-related liver transaminitis (Figure 5). Moreover, the ALT elevations by fully packaged rAAV*EGFP* mixed with PE AAV8 particles that contained more rAAV*hA1AT* genome-bearing virions (40%) were much more pronounced than those with PE AAV8 particles that contained much less rAAV*EGFP* genome-bearing virions (<10%), even though EGFP is much more immunogenic and toxic than hA1AT (Figure 5). In other words, the severity of transaminitis caused by spiked-in PE AAV particles in BALB/c mice appears to be dictated by the proportion of the rAAV genome-bearing virions in the PE AAV8 particles but not the property of the transgene.

The main objective of our current study was to substantiate the negative impact of empty virions on liver-directed gene transduction by rAAV8 but not to elucidate underlining biological mechanisms which is largely unknown at this point. However, as both PE capsids isolated from rAAV8hA1AT and rAAV8EGFP production processes exacerbate transaminitis elicited by rAAV8EGFP liver transduction in BALB/c mice, it is unlikely that the side effects of PE capsids are transgene derived. Among other possibilities, it is plausible that capsid-specific cytotoxic CD8^+^ T cells might have played a role in the total viral particle dose-limiting transaminitis observed in our study. To this end, several recent studies have elegantly addressed the immune biology of empty virions that often contaminates rAAV vector preparations. First, recent studies have suggested that the clinical vector preparations might have contributed to eliciting capsid-specific CD8^+^ T-cell response and transient transaminitis in two patients of the high-dose cohort in a clinical trial using rAAV8 vectors for treatment of hemophilia B.^14,15^ However, the vector used in this trial was isolated by ion exchange chromatography method which cannot separate the full, empty, and PE AAV particles and consisted of a mixture of fully packaged functional, partially packaged nonfunctional, and CE viral particles.^16,17^ It was speculated that the increasing capsid dose by the empty AAV virions could augment the capsid antigen presentation on major histocompatibility complex class I, consequently trigging capsid-specific CD8^+^ T-cell response.^17^ Moreover, in one of these studies,^15^ PE AAV8 and AAV2 virions were collected from the low density band after CsCl gradient ultracentrifugation in the rAAV purification process and characterized for their immune biology in mice; lower but significant proliferative CD8^+^ T-cell responses to these PE AAV particles were indeed detected as compared with fully packaged rAAV particles. In other words, those massively excessive PE particles together with fully packaged vector particles could potentially accentuate cytotoxic capsid-specific CD8^+^ T-cell response and subsequent liver damage. However, the results from this study on residual proliferation of CD8^+^ T cell by PE capsid should be interpreted cautiously as the PE particles with full or partial vector genomes could potentially exacerbate Toll-like receptor 9 signaling.^15,24^ In another interesting *in vitro* study by Li *et al*.^25^, Ova CD8^+^ T-cell epitopes-decorated AAV2 particles were used as a sensitive model system to further characterize cytotoxic capsid-specific T-cell response to the viral particles with and without rAAV genomes. This study suggested defective antigen presentation by CE and Ova CD8^+^ T-cell epitopes-decorated AAV2 virions, which were produced by double transfection in 293 cells without the vector genome plasmid. Importantly, this finding is consistent with our results of which the PE viral particles with more rAAV8*hA1AT* genome-bearing virions (40%) were more immunotoxic than the PE capsids containing less than 10% of rAAV8*EGFP* genome-bearing virions, even though EGFP is known to be much more immunogenic than hA1AT in BLAB/c mice (Figure 5b). Using the similar experimental design, a more recent study by He *et al*.^23^ demonstrated that the pattern and kinetics of capsid antigen cross-presentation from AAV2 and AAV8 transduction *in vivo* are similar.^23^ In sum, all aforementioned studies support our notions that (i) excessive empty capsids are immunotoxic in rAAV liver gene transfer and (ii) PE capsids as a by-product of rAAV production, which contain AAV virions packaged with full or partial viral genomes, are more immunotoxic.

A possible mechanism for the differences between CE and PE viral particles in side effects could be related to structural biology and intracellular trafficking and processing of AAV capsids. It was speculated that CE AAV capsid particles are inefficient in endosome escape and therefore less efficient in capsid antigen cross-presentation.^25^ This hypothesis was further supported by some new insight for structural biology of AAV capsids. In fact, encapsidation of full or partial AAV genomes may lead to capsid conformational changes, the unique N terminus of the capsid VP1 (VP1u) which contains a phospholipase A2 domain, and two nuclear localization signals could be efficiently externalized through fivefold pore.^26^ Such conformational change on capsid structure exposes both phospholipase A2 domain and nuclear localization signal domains and facilitates endosome escape and viral infection.^25^ In contrast, the structural conformation of the CE AAV capsids prevents externalization of unique N terminus of the capsid VP1 in the late endosomes and, therefore, endosome escaping and nucleus trafficking, subsequently resulting in poor antigen presentation. Additionally, inefficient uncoating of CE AAV virions as compared with PE particles could be another possible reason for their lower antigen presentation and immunotoxicity.^25,26^ Nonetheless, further studies may be warranted to elucidate those potential mechanisms.

On the other hand, empty capsids in rAAV preparations may play a beneficial role in overcoming preexisting B-cell immunity to rAAV capsid and enabling rAAV transduction in the patient population that are not acceptable to rAAV gene therapy. This highly innovative concept was clearly demonstrated in a recently report where empty capsids were deliberately added as capsid decoys to the rAAV8 dosing formulation to overcome preexisting humoral immunity against systemically delivered rAAV8,^17^ highlighting its potential clinical benefits in human gene therapy studies. The another novel design of this strategy was to abolish the receptor-binding capability and reserve the neutralizing ability of the empty capsids by site-directed mutagenesis, specifically titrating out host capsid response to rAAV without competing with rAAVs for liver transduction.^17^ Nevertheless, as indicated in the report, this strategy will still face the challenge of crossly presented capsid antigens from overloaded empty capsids, suffering the consequences of adaptive immunotoxicity.^17^ However, the findings from our study might have shed some lights on designing this AAV empty decoy strategy, suggesting that the use of CE instead of PE capsids as decoys will not only overcome host immunity but also potentially reduce cross-presentation of excessive capsids and potential CD8^+^ T-cell response. Therefore, it may be safer and beneficial to add noninfectious mutant CE capsid decoys instead of the PE particles to the therapeutic dosing rAAV vector to blunt the preexisting neutralizing antibodies and reduce potential side effects of capsid decoys in gene therapy recipients. This approach is simple and safe and can be potentially used for vectors derived from all AAV serotypes, which should greatly expand the utilization of AAV vectors in the patients with positive neutralizing antibodies.

In addition, we attempted to investigate whether competition for receptor binding and uptake could indeed be a mechanism for transduction inhibition by empty capsids. Briefly, CE AAV2 capsids were prepared and added to rAAV8*hA1AT* dosing formulations at a REV of 75% to study rAAV8 transduction in C57BL/6 mice. Our preliminary data showed no significant inhibition of rAAV transduction by CE AAV2 virions (data not shown). Another issue that remains unclear with our study is the narrow dose range at which statistically significant inhibition of rAAV8*hA1AT* transduction can be detected in C57BL/6 mice (Figure 3). Some possible reasons for the lack of transduction inhibition of rAAV8 at higher GC doses by AAV8 CE particles may include strong hepatocyte tropism of rAAV8 that results in saturation of liver transduction at higher vector doses as well as the narrow dynamic range of hA1AT enzyme-linked immunosorbent assay that makes it insensitive to detect the impact of CE particles in the high concentration range of serum hA1AT which requires up to 10^5^-fold dilution to fit the standard curves. In fact, this speculation seems to be in alignment with the results in which the transduction repression of rAAV8*nLacZ*, a much less-sensitive cellular reporter gene, was observed in a wide dose range in the liver of the same animal model (Figure 1).

One of the caveats with our study is the use of EM for semiquantitative analysis of the ratios of empty AAV virions in viral preparations. EM technology is very useful and informative for qualitatively studying the structures of virions but not ideal for precise quantitative assessment of empty and full virions in a viral preparation. In addition, EM cannot distinguish full virions packaged with full-length genomes from those with partial viral genomes. Thus, the REVs in the PE AAV8 preparations may be underestimated. These PE particles with partial viral genomes may also contribute to the side effects of the PE virions due to their different capsid conformations as compared with CE particles. In contrast, analytic ultracentrifugation technology is a powerful tool for quantitative characterization of structural heterogeneity of rAAV preparations, allowing precise and selective observation of viral capsid sedimentation in real time.^27,28^ For future studies, it may be necessary to use analytic ultracentrifugation for further characterization of compositions of clinical rAAV lots.

Taken together, this is the first study that systematically investigated the effect of empty virions on rAAV8-medaited transgene expression and viral particle–induced liver damage *in vivo*. Our data not only substantiated the inhibitory effect of empty capsids on rAAV liver transduction but also demonstrated that the source of empty virions not the property of the transgene dictated the severity of viral particle–induced liver transaminitis. Therefore, our results emphasize the necessity to develop and optimize good manufacturing practice–compatible manufacturing process for efficient removal of empty virions from rAAV production/purification process and enhancing therapeutic potency and safety profile of clinical gene therapy vectors. Our findings could also be informative to improving the design and safety of AAV empty capsid decoys to blunt preexisting anti-AAV antibodies in human gene therapy.

## Materials and Methods

### Production of rAAV vectors and empty AAV virions

Preparation and purification of empty virion-free single-stranded rAAV8 vectors expressing secreted human α1-antitrypsin (*hA1AT*) as well as nuclear-targeted *LacZ* (*nLacZ)* and *EGFP* report genes (rAAV8*hA1AT*, rAAV8*nLacZ*, and rAAV*8EGFP*) have been described by Ayuso *et al*.^18^ The transgenes were driven by chicken β-action promoter in these constructs. An AAV8 preparation with 100% complete empty (CE) virions was produced in the same way except for the exclusion of vector genome plasmid (*cis* plasmid) in the transfection step. PE AAV8 virions were produced from the empty virion fractions (low density of ~1.3 g/ml) collected after two steps of CsCl gradient centrifugation in the purification processes for rAAV8*EGFP* and *hA1AT* vectors (high density of ~1.4 g/ml). The REV of viral particles was characterized and semiquantified by high-resolution transmission EM and silver-stained sodium dodecyl sulfate–polyacrylamide gel electrophoresis as previously described.^19^ All the AAV8 vectors and empty virions were produced by the Viral Vector Core at University of Massachusetts Medical School Gene Therapy Center (Worcester, MA).

### Characterization of AAV particles by high-resolution transmission EM and silver-stained sodium dodecyl sulfate–polyacrylamide gel electrophoresis

Morphology of negatively stained AAV virions was characterized by transmission EM in Core Electron Microscopy Facility at the University of Massachusetts Medical School as described previously.^19,29^ In brief, 5 µl of AAV preparation was placed onto a formvar support film and left for 30 seconds. We removed the excess liquid and negatively stained the sample by running six drops of 1% uranyl acetate over the grid to fix and contrast the virus particles. After drying the sample in a controlled humidity chamber, we examined it with a transmission EM and recorded the images. Illustration of the AAV empty particles is primarily exhibited as donut-like shapes formed by the accumulation of the uranyl acetate stain on the dimples.

The purity of AAV preparations was also analyzed by sodium dodecyl sulfate–polyacrylamide gel electrophoresis followed by silver staining as described previously.^30^

### Animal studies

All animal studies were performed under Institutional Animal Care and Use Committee approval by the University of Massachusetts Medical School. Adult C57BL/6 and BALB/c mice (6–8 weeks) were purchased from Harlan Laboratories (Indianapolis, IN). Different vector dosing formulations were administered into C57BL/6 or BALB/c mice intravascularly via tail vein injections. Mouse sera were collected at different time points for detection of A1AT expression and ALT levels. The mouse liver was collected at different time points for detecting GFP and nLacZ expression.

### A1AT reporter gene assay

An enzyme-linked immunosorbent assay–based assay was used to detect serum hA1AT level as described previously.^31^ Rabbit anti-human A1AT antibody and human A1AT standard were purchased from Sigma-Aldrich (St. Louis, MO). Horseradish peroxidase–conjugated goat anit-A1AT antibody was obtained from Fitzgerald (Acton, MA). Other reagents for enzyme-linked immunosorbent assay were purchased from KPL (Gaithersburg, MD). The samples were measured for optical density (OD) at 405 and 600 nm in a luminometer (BioTek Instruments, Winooski, VT). The ΔOD (OD_405_–OD_600_) was used for data analyses, which are based on a linear regression comparison and interpolation of unknown samples against standard dilutions.

### Detection of AAV-mediated transgene expression in liver *in vivo*

Fresh or fixed optimal cutting temperature–embedded liver sections from three hepatic lobes collected from the mock- and vector-injected mice at different postinjection time points were mounted on slides. As described in previous publication,^31^ the EGFP transduction efficiency was measured directly by GFP imaging in fixed sections using a Leica DM 5500B fluorescence microscope made by Leica microsystems (Buffalo Grove, IL). LacZ reporter gene expression on X-gal histochemically stained nonfixed liver sections was examined under a light microscope. Images from five visual fields were analyzed quantitatively using ImageJ analysis software (NIH, Bethesda, MD). Transgene expression was assessed as total area of green fluorescence (GFP) or blue staining (LacZ) (pixel)^2^ per visual field (mean ± SE).

### ALT detection assay

The ALT levels in mouse sera were detected by ALT detection kits purchased from Teco Diagnostics (Anaheim, CA). This colorimetric method for ALT detection is based on dinitrophenylhydrazine formation and is a modification of the classical Reitman Frankel colorimetric end point reaction. The samples were analyzed for OD at 505 nm in a luminometer (BioTek Instruments).

### Statistical analysis

Student’s *t*-test or analysis of variance was used to compare between test results and the control, and they were determined to be statistically significant.
